# Image Enhancement of Computational Reconstruction in Diffraction Grating Imaging Using Multiple Parallax Image Arrays

**DOI:** 10.3390/s20185137

**Published:** 2020-09-09

**Authors:** Jae-Young Jang, Hoon Yoo

**Affiliations:** 1 Department of Optometry, Eulji University, 553, Sanseong-daero, Sujeong-gu, Seongnam-si, Gyonggi-do 13135, Korea; kikijang@naver.com; 2 Department of Electronics Engineering, Sangmyung University, 20 Hongjimoon-2gil, Jongno-gu, Seoul 03015, Korea

**Keywords:** image enhancement, 3-D computational reconstruction, diffraction grating imaging, multiple parallax image arrays

## Abstract

This paper describes an image enhancement method of computational reconstruction for 3-D images with multiple parallax image arrays in diffraction grating imaging. A 3-D imaging system via a diffraction grating provides a parallax image array (PIA) which is a set of perspective images of 3-D objects. The parallax images obtained from diffraction grating imaging are free from optical aberrations such as spherical aberrations that are always involved in the 3-D imaging via a lens array. The diffraction grating imaging system for 3-D imaging also can be made at a lower cost system than a camera array system. However, the parallax images suffer from the speckle noise due to a coherent source; also, the noise degrades image quality in 3-D imaging. To remedy this problem, we propose a 3-D computational reconstruction method based on multiple parallax image arrays which are acquired by moving a diffraction grating axially. The proposed method consists of a spatial filtering process for each PIA and an overlapping process. Additionally, we provide theoretical analyses through geometric and wave optics. Optical experiments are conducted to evaluate our method. The experimental results indicate that the proposed method is superior to the existing method in 3-D imaging using a diffraction grating.

## 1. Introduction

Three-dimensional imaging and sensing for 3-D objects have played an important role in the fields of 3-D data processing, 3-D profiling, 3-D display, and so on [1,2,3,4,5,6,7,8,9]. Acquiring 3-D data is an essential part of 3-D imaging as the first step; thus, various techniques have been studied [1,2,3]. The conventional systems for 3-D imaging are based on a camera array, a lens array, or a moving camera [10,11,12]. Recently, diffraction grating imaging for 3-D imaging was proposed as one of the methods for obtaining parallax images [13,14,15,16], unlike other diffractive imaging [17,18]. The system via diffraction grating imaging consists of an amplitude diffraction grating with a transmissive film, a camera to pick up parallax images, and a laser light source. In diffraction grating imaging, light rays emanating from 3-D objects are diffracted by a diffraction grating. The diffracted rays for the objects can be imaged in the form of an array and a captured version of those parallax images is called a parallax image array (PIA).

A parallax image array containing perspective information of 3-D objects is one of the very efficient storage forms for the 3-D image processing and display fields. Up to date, a camera array, a moving camera, and a lens array have been widely employed for obtaining PIAs [1]. The optical structure of the diffraction grating imaging system is low-cost and low-complex compared to that of the camera array-based system. Diffraction grating imaging has no optical aberrations that are always involved in lens array-based methods, and the captured PIAs can be high-resolution [15,16]. Besides, it has the great advantage of a single optical element used for PIA generation. Thus, a diffraction grating-based imaging system can be one of the promising techniques in 3-D imaging.

However, the diffraction grating imaging has a disadvantage of a small number of parallax images due to the diffraction limit [14]. Additionally, there is a speckle noise problem that occurs in all imaging methods using a laser as a light source [19,20,21]. To solve the problem of the small number of parallax images in diffraction grating imaging, double diffraction grating imaging was studied to increase the number of parallax images [13,14,15,16]. However, no research has been conducted on the reduction of the speckle noise caused by a coherent light source for diffraction grating imaging.

In this paper, we propose a computational 3-D reconstruction method to reduce the speckle noise in diffraction grating imaging via multiple parallax image arrays. The existing computational reconstruction in diffraction grating imaging utilized a PIA to produce a 3-D image [15]. The proposed method employs multiple PIAs to reduce the speckle noise and to enhance the resolution of a 3-D image. The proposed method utilizes the property that the depth of a 3-D object is related to the spatial period of parallax images in each PIA. The spatial period is a parameter for proposed spatial filtering. The spatial filtering for *n* number of PIAs generates *n* number reconstructed images for an object image. Then, those reconstructed images are accumulated to produce an overall 3-D image; thus, this image has reduced speckle noise, increased dynamic range, and enhanced resolution. To demonstrate the practical validity of the proposed method, multiple PIAs for 3-D objects are optically acquired through the proposed diffraction grating imaging. Optical experiments are conducted on multiple PIAs. Additionally, the results are provided to compare our method with the existing method.

## 2. Fundamental Geometric Relationships in Diffraction Grating Imaging

In diffraction grating imaging, scattered lights from a 3-D object are diffracted by a diffraction grating located on the optical path [13,14]. At this time, the diffraction angle of the light rays is determined by the wavelength of the coherent light source in use and the spatial pitch of the grating in use. The diffracted rays are periodically imaged in the form of a 2-D array and this is called a parallax image array. It is seen that the spatial period between parallax images in diffraction grating imaging is proportional to the depth of the 3-D object. Thus, the spatial period between the parallax images increases as the distance between the diffraction grating and the object increases. Considering the optical characteristics such as the image formation position of each parallax image, it is appropriate to view each parallax image as a virtual image. When an object has a three-dimensional volume, it can be observed that these virtual images have their parallax corresponding to the object’s depth and diffraction order. These parallax images have different viewpoints on the object, and they can be captured as a PIA by a pickup device such as a camera. The size and imaging depth of each parallax image are equal to those of the object.

### 2.1. Imaging Position

**Figure 1:**
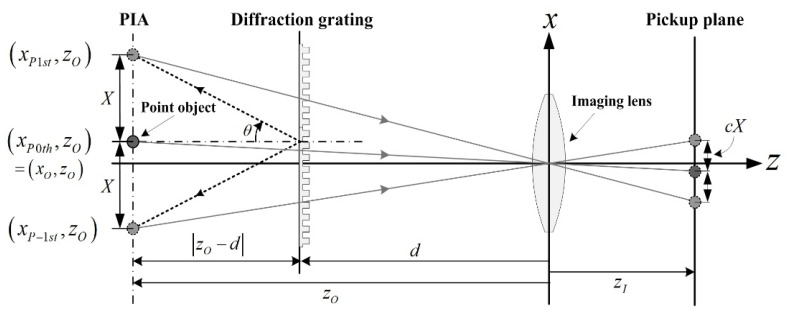
Geometrical relationship between a point object, parallax image array (PIA), and its spatial period *X* in a diffraction grating imaging.

Figure 1 shows the geometrical relationship between the PIA of a point object generated by the diffraction grating and the imaging points where the PIA is imaged by an imaging lens. Here, let the point object be located at (*x_P0th_*, *z_O_*). The *z*-coordinate is *z_O_* for all parallax images. The distance between the diffraction grating and the imaging lens is *d*. In Figure 1, the point object at (*x_O_*, *z_O_*) is associated with the zero-order parallax image at (*x_P0th_*, *z_O_*). The first-order and negative first-order parallax images are located at (*x_P1st_*, *z_O_*) and (*x_P-1st_*, *z_O_*), respectively. They are generated from the corresponding diffraction imaging of the point object. The diffraction angle *θ* is given by *θ* = sin^−1^(*mλ*/*a*) for the diffraction grating, where *m* is the order of diffraction, *λ* is the wavelength of a laser source, and *a* is the pitch of the diffraction grating.

The *x*-coordinate of a parallax image, by considering the location of the object and the diffraction order, is given by

(1) $$x_{Pmth}=x_{O}+\left|z_{O}-d\right|\tan\left(sin^{-1}\left(\frac{m\lambda }{a}\right)\right),$$

where *m* is the order of diffraction and it can be −1, 0, and 1. |*z*_O_−*d*| is a distance between a diffraction grating and an object. *a* is the aperture width of the diffraction grating. Equation (1) implies that the position of the parallax images generated by the diffraction grating is periodic corresponding to the diffraction orders. The geometrical relationship in Equation (1) provides the spatial period of a PIA depending on the object depth in the form of |*x_P_*_(*s*)*th*_ − *x_P_*_(*s*-1)*th*_|, for *s* = 0 or 1. The spatial period is then rewritten by

(2) $$X_{z_{O}}=\left|z_{O}-d\right|\tan\left(sin^{-1}\left(\frac{\lambda }{a}\right)\right).$$

### 2.2. Parallax Angle

**Figure 2:**
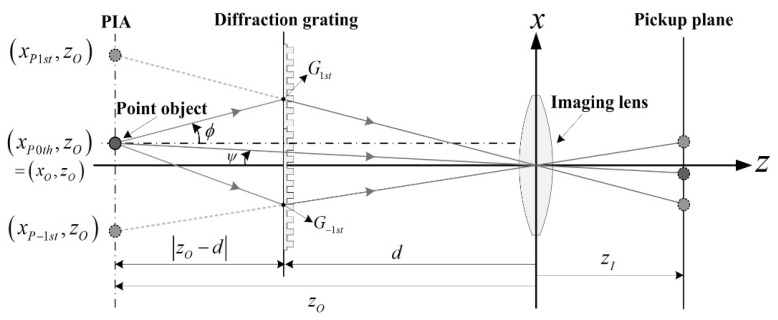
Parallax angle of a point object, *ϕ*, corresponds to the 1st order parallax image.

Figure 2 shows the geometric relationship to determine the parallax angle of a point object. The *z*-location of parallax images is generated by diffraction grating imaging and is the same as that of the point object. Although the rays that reach the imaging plane seem to come from parallax images as described in Figure 1, only the rays emanating from the object are real. The parallax angle of the object corresponding to each parallax image can be then explained by analyzing the relationship between the light rays from the object and the virtual rays from the parallax image.

Figure 2 shows the geometric relationship among the positions of parallax images generated by a diffraction grating, the chief ray path of the point object, and the virtual ray path of its parallax images. The parallax angle of each parallax image is depicted in Figure 1. Here, the virtual rays going to the optical center of the lens coming from the first-order (1st) and negative first-order (−1st) parallax images meet the diffraction grating at point *G*_1__st_ and *G-*_1__st_, respectively. At the points *G*_1__st_ and *G-*_1__st_, the paths of the real rays from the point object are redirected to the optical center of the imaging lens. Consequently, the parallax angle *ϕ* of the point object corresponding to the *mth* order parallax image is given by

(3) $$\phi_{mth}=\tan^{-1}\left(\frac{G_{mth}-x_{O}}{\left|z_{O}-d\right|}\right),$$

where *G_mth_* in Equation (3) is given by

(4) $$G_{mth}=\left(\frac{d}{z_{O}}\right)x_{Pmth}.$$

The parallax for each parallax image is determined by the parallax angle *ϕ* and the angle *ψ* between the imaging lens and the object, as shown in Figure 2.

## 3. Wave Optical Analysis of Imaging Formation in Diffraction Grating Imaging

The optical characteristics of a PIA in diffraction grating imaging can be represented using an impulse response and scaled version of object intensity by the use of the periodic property of a PIA depending on the depth of an object. In conventional 2-D imaging, the intensity function *g*(*x_P_*) can be calculated as *g*(*x_P_*) = *h*(*x_P_*) ∗ *f*(*x_P_*), where ∗ means the convolution operation, *x_P_* is the *x* coordinate on a PIA, *h*(*x_P_*) is the impulse response, and *f*(*x_P_*) is a function of object intensity.

Meanwhile, the image intensity for 3-D objects can only be localized at the plane *z_O_* such that the image intensity is written as *g*(*x_P_*)|*_zo_* = *f*(*x_P_*)|*_zo_* ∗ *h*(*x_P_*)|*_zo_*. Note that the *z_O_* dependence is because the impulse response for intensity is dependent on the object intensity on the depth *z_O_*. Considering the continuously distributed intensity of 3-D objects, the *z_O_* dependent image intensity can be given by

(5) $$g\left(x_{P}\right)=\int h\left(z_{O},x_{P}\right)*f\left(z_{O},x_{P}\right)dz_{O},$$

where the intensity *g*(*x_P_*) means a linear sum of image intensity. Here, the intensity impulse response *h*(*z_O_*, *x_P_*) in Equation (5) can be approximated by an array of *δ*-functions. The intensity impulse response *h*(*z_O_*, *x_P_*) in Equation (5) is written by *h*(*z_O_*,*x_P_*)=∑*δ*(*x_O_*-*nX*), from Equations (1) and (2), where *X* is calculated from Equation (2). The intensity impulse response can be thus given by

(6) $$h\left(z_{O},x_{P}\right)=\sum_{n=-1}^{1}\delta \left(x_{O}-nX_{z_{O}}\right).$$

Here, it is seen that the intensity impulse response in diffraction grating imaging can be represented by a *δ*-function array where the spatial period depends on a given depth of 3-D objects [9].

Next, we consider a scaled version of the object intensity function *f*(*z_O_*, *x_P_*) in Equation (5). The average intensities of parallax images are different since the divided energies of rays in a diffraction grating are different. Thus, a weighted version of intensity function is required to express the intensity function accurately, which is defined by

(7) $$f\left(z_{O},x_{P}\right)=\left|\frac{z_{I}}{z_{O}}\right|f_{O}\left(z_{O},-x_{O}\right),$$

where *f_0_*(*z_O_*, *x_P_*) denotes the object intensity function of the zero-order parallax image. Thus, the intensity of a PIA can be derived by substituting Equations (6) and (7) into Equation (5), and it is given by

(8) $$g\left(x_{P}\right)=\iint \sum_{n=-1}^{1}\delta \left(x_{O}-nX_{z_{O}}\right)\left|\frac{z_{I}}{z_{O}}\right|f_{O}\left(z_{O},-x_{O}\right)dx_{O}dz_{O}.$$

This implies that the intensity *g*(*x_P_*) is a periodic function in diffraction grating imaging and it is continuous since the object intensity is continuous in all directions of the 3-D object space.

## 4. Computational 3-D Reconstruction with Multiple Parallax Image Arrays

In general, existing computational reconstruction methods of a 3-D image from a PIA in 3-D imaging are based on the back-projection method, where each 2-D parallax image is projected on the 3-D space. The projected image expands continuously as the distance increases. Projecting all parallax images on the 3-D space provides some object area in the parallax images to overlap each other at a specific depth. This process can be conducted at any 3-D location; thus, a 3-D image is reconstructed. Additionally, the more parallax images that are engaged in back-projection, the better the acquired quality is. However, the existing diffraction grating imaging uses a small number of parallax images; for example, 3 × 3 parallax images in a PIA. The reconstructed 3-D image may suffer from the speckle noise of a laser source. Moreover, an accurate method of extracting individual parallax images from a PIA is required because there is no apparent boundary between parallax images in a PIA.

In this paper, we propose a computational reconstruction method with multiple parallax image arrays in diffraction grating imaging. The proposed method consists of a pickup process of multiple PIAs by moving a diffraction grating axially and a computational reconstruction process with these multiple PIAs. To capture multiple PIAs for the proposed method, we apply a moving stage to our previous system for diffraction grating imaging to axially move a diffraction grating plate between objects and the camera in use. To reconstruct a 3-D image from the multiple PIAs captured by our pickup process, we propose spatial-filtering on each PIA, using a delta function array to reduce the speckle noise. Here, our computational reconstruction for a 3-D image is performed by estimating the period of each PIA corresponding to a specific depth, considering the property that the object is periodically imaged corresponding to the object depth.

As analyzed above, the distance between the individual parallax images in a PIA increases as the depth of the object moves away from the diffraction grating. Thus, a 3-D image of a specific depth can be reconstructed by convolving a PIA with a *δ*-function array, where the spatial period depends on the desired depth [22]. Consequently, the spatially filtered PIA at a target depth *z_O_* is given by

(9) $${R\left(x_{P}\right)|}_{z_{O}}=\frac{1}{N}g\left(x_{P}\right)*\sum_{n=-1}^{1}\delta \left(x_{O}-nX_{z_{O}}\right),$$

where *X_zO_* is the spatial period for a target depth and also *N* is the total number of parallax image arrays.

**Figure 3:**
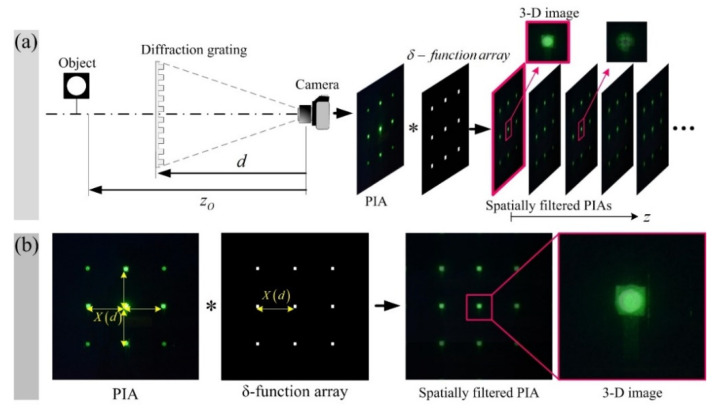
3-D image reconstruction process with single PIA. (**a**) PIA acquisition process and spatial filtering process. Sequential depth sliced images of the object space are generated through the convolution of the PIA and the *δ-*function array whose spatial period is continuously changed. (**b**) The spatial filtering result and the 3-D reconstruction image when the parallax image and the *δ-*function array have the same spatial period.

Figure 3 is intended to illustrate Equation (9) and shows the PIA pickup process and the spatial filtering process for a single PIA. The left side of Figure 3a shows the PIA acquisition process, where the distances of the object and the diffraction grating from the camera are *z_O_* and *d*, respectively. The PIA obtained in this process corresponds to *g*(*x_P_*) in Equations (8) and (9). The right side of Figure 3a shows the spatial filtering process using the convolution of a PIA and a *δ-*function array. In this process, the spatial period of the *δ-*function array is sequentially changed corresponding to the depth of the object space, and as a result, spatially filtered PIAs corresponding to the depth are sequentially generated. As mentioned above, the spatially filtered PIA can be expressed by Equation (9). Figure 3b shows the result of spatial filtering for the case, where the spatial periods of the PIA and the *δ-*function array are the same.

**Figure 4:**
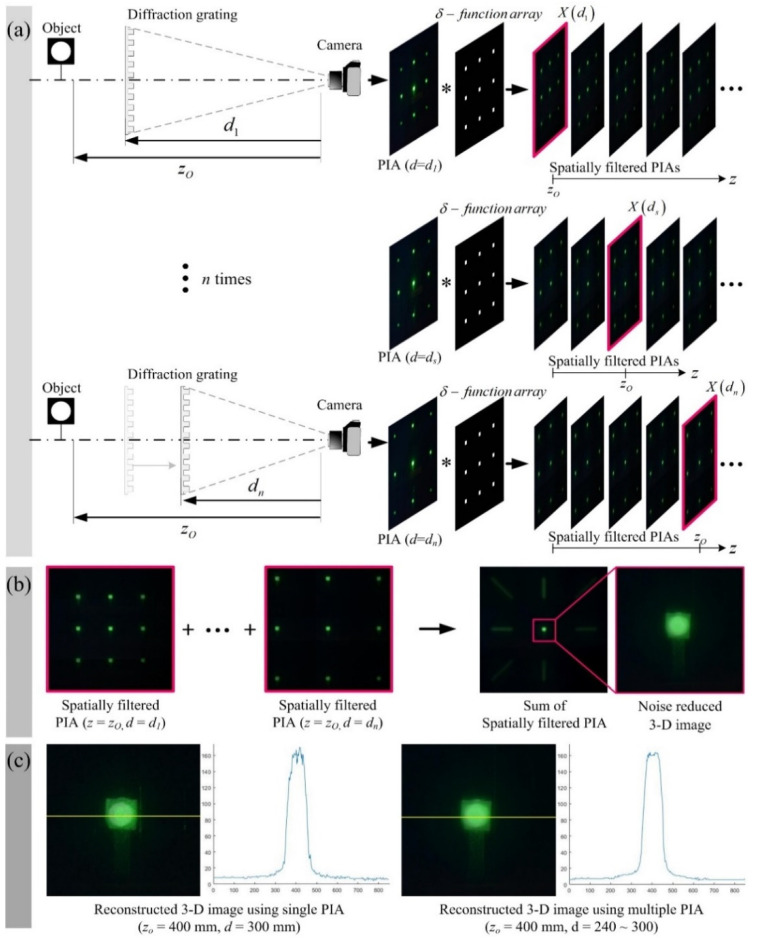
3-D image reconstruction process with multiple PIAs. (**a**) PIA acquisition process and spatial filtering process. The distance *d* between the diffraction grating and the camera is sequentially changed to acquire PIAs. Spatial filtering is performed on each PIA to extract PIAs corresponding to successive depths. (**b**) After adding spatially filtered PIAs corresponding to the same depth, *z_O_*, the proposed 3-D image is reconstructed. (**c**) Comparison of 3-D reconstructed images in intensity profile.

Figure 4 shows the proposed method of reconstructing a 3-D image using multiple PIAs. The left side of Figure 4a shows the process of acquiring multiple PIAs. During the PIA acquisition process, the distance between the diffraction grating and the camera is adjusted sequentially from *d_1_* to *d_n_* to acquire a group of *n* PIAs. According to Equations (2) and (3), the spatial period and parallax angle of the obtained PIA increase as *d* decreases. In the spatial filtering process, spatial filtering is performed for each of the PIAs with different spatial periods for the same object. Since the spatial period of the PIA depends on *d*, the spatial period for each PIA can be expressed as *X*(*d*). The spatially filtered PIA corresponding to the depth of the object is extracted through the convolution between the PIA and the *δ-*function array having the same spatial period, *X*(*d*). In Figure 4a, as an example of this, when the position of the diffraction grating is *d*, it is indicated by a red line on the border of the spatially filtered PIA. Figure 4b shows that the 3-D image with reduced noise is reconstructed by summing spatially filtered PIAs. Here, the spatially filtered PIAs are extracted for the same depth from each of the original PIAs. Therefore, the proposed 3-D image reconstruction method can be expressed by

(10) $${U\left(x_{P}\right)|}_{z_{O}}=\frac{1}{n}\sum_{k=1}^{n}{R\left(x_{P},\;d_{k}\right)|}_{z_{O}},$$

where *n* is the total number of PIAs.

Figure 4c shows 3-D images reconstructed by the conventional and proposed methods and their intensity profiles, respectively. Our diffraction grating imaging can acquire as much data as desired to reconstruct an object image. Therefore, the superposition of multiple PIAs enables ray energy from 3-D objects to be concentrated in a specific depth; a random noise such as the speckle noise can be suppressed in our method, as shown in Figure 4c. Additionally, our computational reconstruction method with multiple PIAs can provide more dynamic range and entropy; thus, it can support high-resolution imaging in diffraction grating imaging.

## 5. Optical Experiments and Discussion

**Figure 5:**
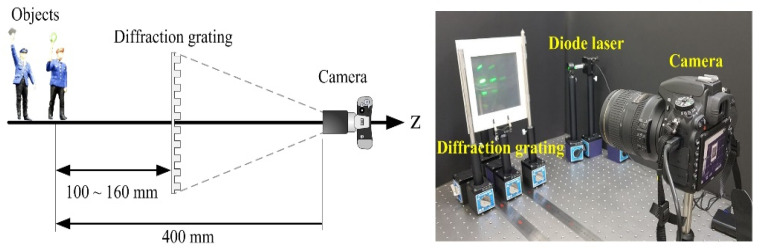
Experimental setup for our pickup process in diffraction grating imaging. The distance between the camera and the closest object is fixed at 400 mm.

Optical experiments with multiple PIAs using a diffraction grating are conducted to verify the theoretical analysis described above and to evaluate the proposed method. The proposed computational reconstruction method with multiple PIAs is performed to compare with the previous method with a PIA. Our experimental setup for the PIA pickup of multiple PIAs, as shown in Figure 5, is based on a moving diffraction grating. In the process of obtaining PIAs, the distance between the camera in use and the closest object is 400 mm. The initial distance is 100 mm away from the closest object. By moving the diffraction grating toward the camera, distances change from 100 to 160 mm with an increment of 10 mm. A total of seven PIAs are captured according to the distances between the diffraction grating and the object. Two diffraction gratings, attached perpendicularly to each other, are used in our experiment. Each diffraction grating has a spatial resolution of 500 lines/mm. For illuminating the objects, a laser source with a wavelength of *λ* = 532 nm is employed.

**Figure 6:**
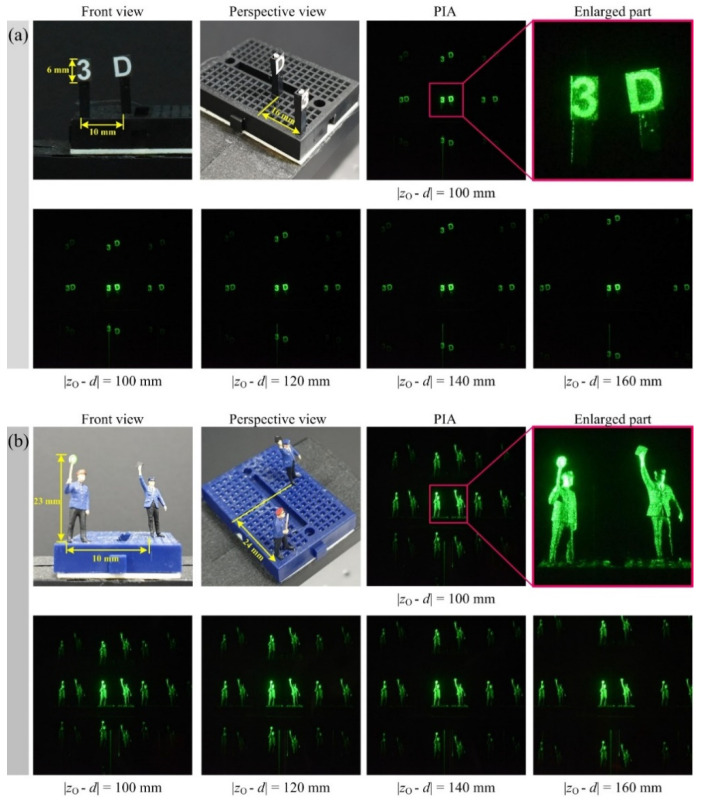
Objects used in the PIA pickup process and captured PIAs. Four PIAs among the seven PIAs acquired according to the distance between the diffraction grating and the object are displayed. (**a**) 3-D objects of letters of ‘3’ and ‘D’. An example of the captured PIA and an enlarged image of the center portion of the PIA; (**b**) 3-D objects of male models. An example of the captured PIA and an enlarged image of the center portion of the PIA.

Figure 6 shows views of the front and perspective of the object and its parallax image arrays captured by our diffraction grating imaging. Two sets of 3-D objects are utilized to carry out the optical experiments and to evaluate the proposed computational reconstruction method. The letters of ‘3’ and ‘D’, as shown in Figure 6, are used as plane-shape objects and two male models are also employed as 3-D volume objects. Two examples of PIAs captured by our pickup process and their enlarged versions are shown in Figure 6a,b, where the strong speckle noise exists. Each bottom of Figure 6a,b shows four PIAs of the total seven PIAs according to the distance |*z_O_* − *d*| between the diffraction grating and the nearest object. Each PIA in Figure 6 has a resolution of 3007 × 3007 pixels, and 3 × 3 parallax images are in each PIA.

It is seen that the intensities of parallax images are different due to the efficiency of a diffraction grating. The efficiencies of the diffraction grating in use are approximately 85% and 50% for the zero-order and the first-order diffraction, respectively. However, our computational reconstruction method has robustness against this intensity difference since our reconstruction method accumulates all parallax images that are split by the diffraction grating. Thus, diffraction efficiency for a diffraction grating does not matter in our 3-D computational reconstruction method.

**Figure 7:**
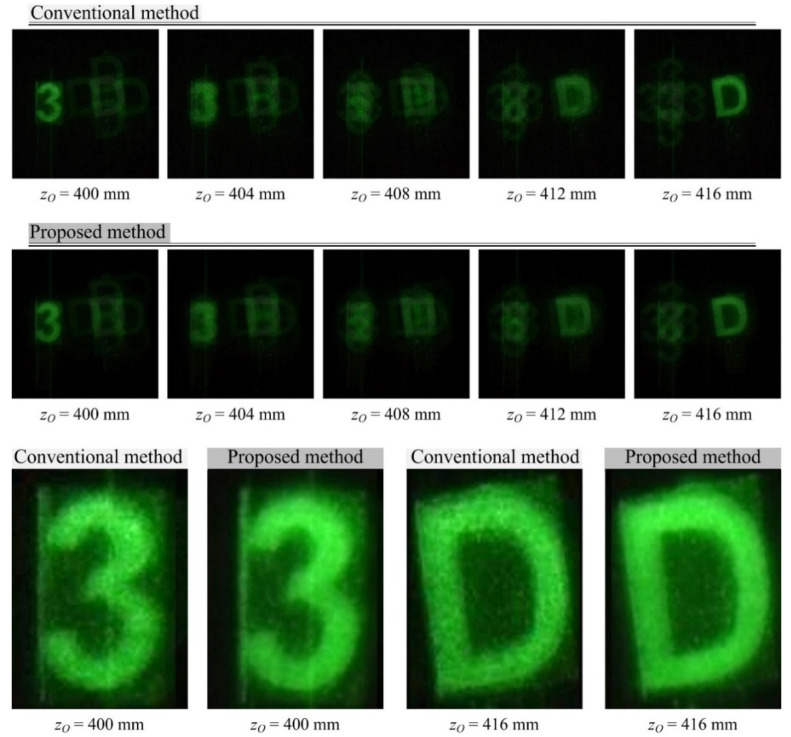
The 3-D computational reconstruction image by each of the conventional and proposed methods for the object in Figure 6a. The distance between the camera and the reconstruction plane is indicated at the bottom of each reconstructed image. At the bottom, zoomed images are displayed with normalized intensity.

Figure 7 shows 3-D computational reconstruction results for the objects in Figure 6a, comparing the proposed method with the conventional method in diffraction grating imaging. In the existing computational reconstruction method, the PIA according to the distance of 100 mm away from the objects is used as an input PIA. The spatial period of the *δ*-function in the reconstruction process is set by the depth of the reconstruction plane along the *z*-axis, as described in Equation (9). The number presented at the bottom of each reconstructed image is the distance between the reconstruction plane and the camera. In the proposed method, seven PIAs according to the distance between the objects and the diffraction grating are used as input PIAs. The computational reconstruction of the image corresponding to each depth is described in Figure 4. The bottom of Figure 7 shows the zoomed versions of the plane images at 400 and 416 mm which are reconstructed by the conventional and proposed methods. For a fair visual comparison, the zoomed version is normalized in intensity by using

(11) $$R_{ij}=255\frac{R_{ij}}{\max R_{ij}},$$

where *R_ij_* is a pixel value of a reconstructed image and the image contrast is normalized for the reconstructed images from the previous and proposed method. It is seen that the speckle noise was significantly reduced by the proposed method, compared with the existing method. Additionally, the image edges from our method are much sharper than those from the existing method. Therefore, image resolution is enhanced by the proposed method.

**Figure 8:**
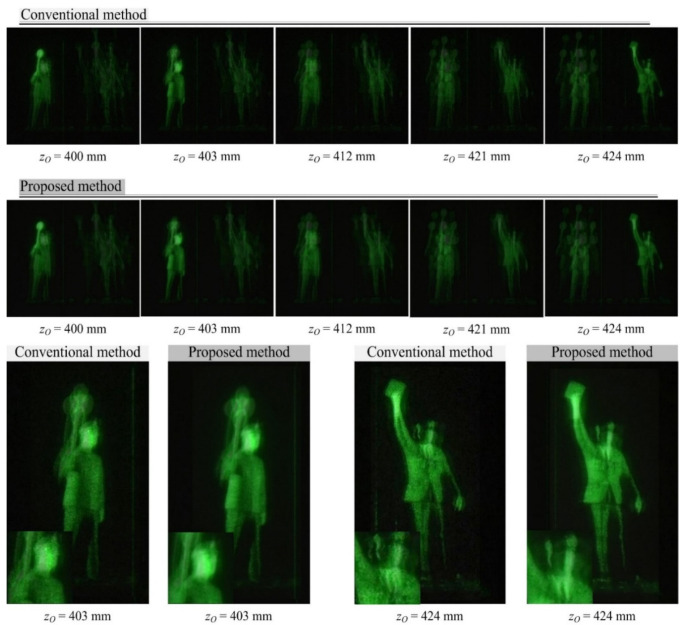
The 3-D computational reconstruction image by each of the conventional and proposed methods for the object in Figure 5b. The distance between the camera and the reconstruction plane is indicated at the bottom of each reconstructed image. At the bottom, zoomed images are displayed with normalized intensity.

Figure 8 shows 3-D computational reconstruction results for the objects in Figure 6b using the conventional method and the proposed method, respectively. The experimental setup is the same as the description for Figure 7. The bottom of Figure 8 shows the object images and their enlarged portions at the lower-left corners for the depths of 403 and 424 mm. Here, the zoomed version is normalized in intensity based on Equation (11), as discussed in Figure 7. The conventional method produces the resulting images with the speckle noise, whereas the proposed method suppresses the speckle noise significantly. Especially, the two reconstructed objects located at *z_o_* = 424 mm show that the proposed method provides much sharper image edges than the previous method, by inspection of the neck area of the reconstructed object. Therefore, the visual comparison confirms image enhancement for computational 3-D reconstruction using multiple parallax image arrays in diffraction grating imaging.

To evaluate the proposed method objectively, we introduce two measures such as dynamic range and entropy since the original signal is not available in optical experiments. The dynamic range is defined as the difference between maximum intensity and the minimum intensity of a reconstructed image. It is important in measuring image contrast. Additionally, the entropy is defined as the average of information per sample such that entropy = −Σ(*p_i_*)^−1^ × log(*p_i_*). Here, *p_i_* is the probability of the intensity value of a pixel. It can be a measure to determine how much information is in a reconstructed image. To compare our method with the previous method, four object images are extracted such as ‘3’, ‘D’, ‘Front man’, ‘Rear man’, as shown in Figure 7 and Figure 8. Table 1 indicates the results from both methods in terms of dynamic range and entropy. The dynamic range of the proposed method is wider than the previous method because seven reconstructed images from seven PIAs are accumulated into a reconstructed image with a wide dynamic range. It is seen that the average dynamic range of the previous method is around 161.8, which means a reconstructed image from the previous method is possibly dark and it needs a brightness control. Here, the speckle noises can be stronger due to the limited dynamic range, as shown at the bottoms of in Figure 7 and Figure 8. On the other hand, the dynamic range of the proposed method is larger enough to control the brightness and more information can be extracted than the previous method while suppressing the speckle noise.

In addition, the higher entropy of a reconstructed image from our method is obtained because of using multiple PIAs. For example, the average entropy of reconstructed images from our method is around 7.80 bit/pixel. This is an improvement of 50.5%, compared with the average entropy of 5.15 bit/pixel from the existing method, as shown in Table 1. Generally, the image entropy increases when random noise such as the speckle noise is embedded. On the other hand, the proposed method provides much higher entropy of the reconstructed images although it reduces the speckle noise a lot.

## 6. Conclusions

In this paper, we proposed a computational reconstruction method for 3-D images with multiple parallax image arrays in diffraction grating imaging. The more parallax images are engaged in 3-D computational reconstruction, the less speckle noise is shown in the reconstructed images, according to our optical experimental results. Additionally, the image edges of the reconstructed image from our method are much sharper than that of the existing method. Therefore, the proposed method enhanced the image quality of 3-D images in diffraction grating imaging. This result indicates that computational reconstruction via diffraction grating imaging can be applied to many applications.

## Figures and Tables

**Table 1:** Experimental results of objective measures for image enhancement.

Test Objects	Dynamic Range		Entropy (bit/pixel)		Note
	Previous	Proposed	Previous	Proposed	
3	148	896	5.96	8.54	Plane
D	145	846	5.84	8.41	Plane
Front man	204	1313	4.57	7.28	Real 3-D
Rear man	150	981	4.29	6.96	Real 3-D
Ave.	161.8	1009	5.17	7.80	
